# Prophylactic Fixed-Rate Phenylephrine Versus Norepinephrine Infusion in the Prevention of Post-spinal Anesthesia Hypotension During Cesarean Delivery

**DOI:** 10.7759/cureus.41251

**Published:** 2023-07-01

**Authors:** Anisha Pauline, K Arthi, Aruna Parameswari, Mahesh Vakamudi, Akilandeswari Manickam

**Affiliations:** 1 Anesthesiology, Sri Ramachandra Institute of Higher Education and Research, Chennai, IND

**Keywords:** cesarean section, post-spinal hypotension, spinal anesthesia, vasopressor infusion, phenylephrine, norepinephrine

## Abstract

Background

Maternal hypotension following spinal anesthesia can be actively countered by the use of vasopressors. Prophylactic infusion of vasopressors with a rescue bolus dosing was observed to be more effective for hemodynamic stability when compared to administering a bolus dose alone. Although phenylephrine is the recommended drug to treat spinal hypotension, many recent studies have focussed on the role of norepinephrine infusions during cesarean section. In this study, we compared prophylactic fixed-rate intravenous infusions of phenylephrine and norepinephrine during cesarean delivery under spinal anesthesia and the requirement of intraoperative provider-administered rescue bolus of phenylephrine needed to overcome post-spinal anesthesia hypotension.

Methodology

A total of 208 patients undergoing elective cesarean section under spinal anesthesia were randomly assigned to two groups (group P and group N). Group N included 104 patients who received norepinephrine infusion at a rate of 2.5 μg/minute (0.04 μg/kg/minute), and group P included 104 patients who received phenylephrine infusion at a rate of 50 μg/minute (0.8 μg/kg/minute) to treat spinal hypotension. The primary outcome of our study was to compare the reduction in the number and total dose of intraoperative provider-administered rescue bolus of phenylephrine needed to maintain systolic blood pressure. The secondary outcome of our study was to compare the neonatal outcome using umbilical venous blood gas sampling and Apgar score at one and five minutes.

Results

The total number of phenylephrine rescue bolus required to treat hypotension was significantly lower in group N (p = 0.0005) compared to group P. The neonatal outcome was similar between the two groups.

Conclusions

Prophylactic norepinephrine infusion when compared to prophylactic phenylephrine infusion is associated with a lesser requirement of rescue phenylephrine boluses.

## Introduction

Spinal anesthesia is the preferred technique for an elective cesarean section as it avoids airway manipulation and the complications of general anesthesia [1]. However, if not actively prevented by pharmacological measures, it causes maternal hypotension in most women, with the incidence being as high as 60% [2,3]. The use of vasopressors is the most reliable method of counteracting hypotension and is highly recommended as prolonged maternal hypotension can lead to serious side effects such as nausea, vomiting, cardiovascular instability, decreased blood flow to the uteroplacental complex, and consequent fetal acidosis [4].

The most commonly used vasopressors are phenylephrine and ephedrine. Ephedrine is associated with maternal tachycardia and decreased fetal pH [5]. Phenylephrine has been reported to be accompanied by a dose-related decrease in maternal heart rate (HR) and the subsequent fall in cardiac output [6]. Recently, norepinephrine has been proposed as an alternative vasopressor in obstetric anesthesia owing to its dual α- and β-agonist activity [7,8].

Prophylactic infusion of vasopressors with a rescue bolus dosing was observed to be more effective for hemodynamic stability when compared to administering a bolus dose alone. The advantages included reducing the workload of clinicians while providing increased maternal comfort [9]. In this study, we compared norepinephrine with phenylephrine infusion in the treatment of post-spinal anesthesia maternal hypotension. The primary outcome was to observe which of the two vasopressor infusions reduced the number and the total dose of intraoperative provider-administered rescue bolus of phenylephrine needed to maintain systolic blood pressure (SBP). Umbilical venous blood gas parameters such as pH, pCO_2_, pO_2_, HCO_3_, and neonatal Apgar scores at one and five minutes were the secondary outcomes. We hypothesized that a prophylactic fixed-rate infusion of norepinephrine would require lower intraoperative bolus doses to maintain SBP in comparison to phenylephrine.

## Materials and methods

This prospective, randomized, double-blind, controlled study was conducted after obtaining institutional ethics committee approval with prospective registration with the Clinical Trials Registry of India (CTRI/2019/08/020995). The study was conducted from August 2019 to February 2020. Written informed consent was obtained from 208 pregnant women belonging to the American Society of Anesthesiologists (ASA) grade II with uncomplicated normal singleton beyond 36 weeks term pregnancy who were scheduled for elective lower-segment cesarean section under spinal anesthesia.

Patients with absolute/relative contraindications to spinal anesthesia such as allergy to any of the study drugs; body mass index beyond 40 kg/m^2^; emergency cesarean; multiple gestations; gestational hypertension; gestational diabetes mellitus; cardiovascular, cerebrovascular, or renal disease; conditions which led to a reduction in blood flow to the placenta such as oligohydramnios and intrauterine growth restriction; and patients suffering from connective tissue disorders were excluded from the study. A complete preoperative evaluation was done on the previous day of the procedure, and necessary investigations were done according to the ASA guidelines. Standardized institutional guidelines for fasting and antacid premedication were followed.

**Figure 1:**
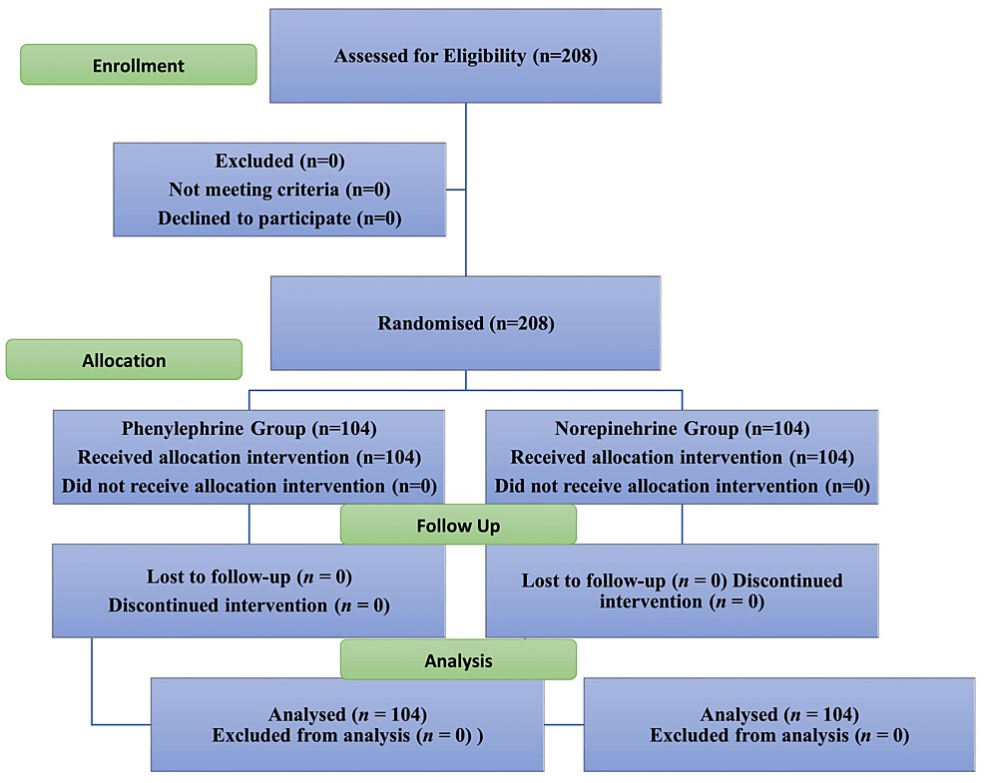
Consolidated Standards of Reporting Trials (CONSORT) diagram showing the process flow.

On the day of the surgery, the patients were randomly assigned to either of the two study groups by computer-generated randomization codes placed in sealed and sequentially numbered envelopes. The phenylephrine group had 104 patients, and the norepinephrine group had 104 patients. (Figure 1) The investigator and the patient were both blinded to the study drugs. The infusions were prepared in 50 mL syringes by an anesthetist not participating in the study and labeled as group P or group N.

Upon arrival at the operation theater, two large bore peripheral intravenous cannulas were placed (one for fluids and the other for vasopressor infusion). Baseline HR, SBP, and diastolic blood pressure (DBP) were noted. Spinal anesthesia was administered using a 27-gauge Pencan needle in a sitting position at the L3-L4 space. Hyperbaric bupivacaine 10 mg and Fentanyl 20 μg with a total volume of 2.4 mL were administered intrathecally.

To standardize and study the effects without affecting the potency of the drugs, vasopressor doses were taken in an equipotent ratio (20:1) based on previous studies [10-16]. In group P, phenylephrine infusion was initiated at a rate of 50 μg/minute (0.04 μg/kg/minute) or 60 mL/hour (1 mL/minute). The infusion was prepared by taking 2 mg of phenylephrine and diluting it with 0.9% normal saline (NS) to attain a total volume of 40 mL and a concentration of 50 μg/mL. In group N, Norepinephrine infusion was prepared by taking 0.1 mg of norepinephrine and diluting it with 0.9% NS to attain a total volume of 40 mL and a concentration of 2.5 μg/mL. This was initiated at a rate of 2.5 μg/minute (0.8 μg/kg/minute) or 60 mL/hour (1 mL/minute) [17]. Both vasopressor infusions were started at the same time that cerebrospinal fluid was obtained before the injection of the local anesthetic into the cerebrospinal fluid. An independent blinded anesthetist who was posted in the theater managed the infusions and collected the data for analysis during the cesarean delivery.

After an intrathecal injection was given, patients were placed in a supine position and a wedge was placed beneath the right hip which gave a 20-degree left tilt. The spinal sensory level was checked bilaterally by cold sensation. Hemodynamic variables such as HR, non-invasive blood pressure, and SpO_2_ (oxygen saturation) were analyzed at different time intervals (baseline, five minutes, 10 minutes, 20 minutes, 30 minutes, 45 minutes, 60 minutes, 75 minutes, 90 minutes, and 120 minutes) after starting the infusion.

In this study, post-spinal maternal hypotension was defined as a decrease in SBP ≤20% of baseline values or less than 90 mmHg and was dealt with a rescue bolus of phenylephrine 20 μg [17]. Hypertensive episodes were defined as SBP 20% higher than baseline values after the use of the vasopressor infusion and were managed by discontinuing the vasopressor infusion excluding the patients from the study [17]. An HR lower than 60 beats per minute indicated bradycardia and was treated by stopping the infusion. Bradycardia accompanied by hypotension was treated with a bolus of ephedrine 6 mg. If bradycardia was resistant to ephedrine, then atropine 0.6 mg was administered and patients were excluded from the study.

Immediately after delivery when the surgeon applied double clamps to the umbilical cord, an umbilical venous sample was taken in a heparinized syringe from the clamped segment of the umbilical cord and sent for blood gas analysis immediately. Neonatal outcome was assessed by Apgar scoring at one and five minutes by a pediatrician blinded to the study. Other complications such as maternal nausea, vomiting, and shivering were observed.

The infusion was continued till the end of the procedure and stopped. Patients were monitored in the operating room for 10 minutes and shifted to the post-anesthesia care unit (PACU). In the PACU, patients were monitored for one hour and shifted to the ward.

The primary outcome was to observe which of the two vasopressor infusions had a reduction in the number and the total dose of intraoperative provider-administered rescue bolus of phenylephrine needed to maintain SBP. Umbilical venous blood gas parameters such as pH, pCO_2_, pO_2_, HCO_3_, and neonatal Apgar scores at one and five minutes were the secondary outcomes.

The sample size was calculated using the nMaster software version 2.0 based on the primary outcome of the number of rescue bolus doses of phenylephrine to prevent spinal hypotension. In a previous study by Vallejo et al. [18], the proportion of patients who required rescue boluses was group phenylephrine at 65.8% (n = 25) versus group norepinephrine at 48.8% (n = 21). With this information, to achieve a mean difference of 0.17 in the number of rescue bolus doses of phenylephrine between the groups and with 80% power of the study and an alpha error of 5%, we estimated a minimum sample of 186 cases (93 per group). To account for those who might be excluded from the study due to various reasons, an additional 11 cases in each group were included for a final sample size of 208 cases (104 per group).

The statistical analysis was done using SPSS for Windows version 22.0 (IBM Corp., Armonk, NY, USA). Discrete or categorical data were presented in numbers or as percentages (%). Continuous data were presented as mean/standard deviation (SD). Normality was checked using the Kolmogorov-Smirnov test and compared using Student’s t-test (unpaired). The categorical data were compared using the chi-square or Fisher’s exact test, whichever was applicable. Student’s t-test was used to compare hemodynamic responses, and the trend within each group over time was analyzed using repeated-measure analysis of variance (ANOVA). P-value <0.05 was considered significant.

## Results

A total of 208 female patients undergoing elective lower-segment cesarean surgery under spinal anesthesia participated in this study. The distribution of age was comparable between the two groups. There was no statistically significant difference in age between the two groups (p = 0.614) (Table 1).

**Table 1:** Comparison of patient characteristics. The data are represented as mean (SD).

Parameters	Phenylephrine group (n = 104)	Norepinephrine group (n = 104)	P-value
Age (in years)	28.4 (4.31)	28.12 (3.91)	0.61

Norepinephrine group patients required less bolus dosing compared to the phenylephrine group. Of the total 208 patients, 36 patients in group P required one bolus of phenylephrine and 13 patients required two boluses of phenylephrine, whereas in group N only 23 patients required one bolus of phenylephrine and none of them required two boluses of phenylephrine to maintain SBP. There was less requirement for rescue bolus in group N compared to group P (p = 0.0005) (Table 2).

**Table 2:** Comparison of the number of phenylephrine boluses required between the groups. **: P-values less than 0.01 were considered highly significant.

Parameters	Phenylephrine group (n = 104)	Norepinephrine group (n = 104)	P-value
Nil bolus	55 (52.9%)	81 (77.9%)	0.0005**
Single bolus dose	36 (34.6%)	23 (22.1%)	
Two bolus doses	13 (12.5%)	0 (0%)	

The blood gas analysis of neonates was done through umbilical cord blood sampling. The mean umbilical cord venous blood pH values were similar in both groups, with 7.32 ± 0.03 in the phenylephrine group, and 7.33 ± 0.03 in the norepinephrine group. The difference between the two groups was statistically insignificant (p = 0.062). Umbilical cord venous pCO_2_ values were similar in both groups and were found to be statistically insignificant (p = 0.29). The mean ± SD value in the phenylephrine group was 45.91 ± 5.84 and in the norepinephrine group was 45.12 ± 5.10 (Table 3).

**Table 3:** Comparison of umbilical cord blood gases and neonatal outcomes. The data are represented as mean (SD). P-values less than 0.05 were considered significant.

Parameters	Phenylephrine group (n = 104)	Norepinephrine group (n = 104)	P-value
Venous pH	7.32 (0.03)	7.33 (0.03)	0.062
Venous pCO_2_	45.91 (5.84)	45.12 (5.10)	0.29
Venous pO_2_	23.95 (5.63)	25.28 (6.28)	0.10
Venous HCO_3_	24.29 (2.35)	24.30 (2.44)	0.98

The mean umbilical cord venous pO_2_ was 23.95 ± 5.63 in the phenylephrine group and 25.28 ± 6.28 in the norepinephrine group. The difference between the two groups was statistically insignificant (p = 0.10). Umbilical cord HCO_3_ values were similar in both groups and were statistically insignificant (p = 0.98). The mean ± SD in the phenylephrine group was 24.29 ± 2.35 and in the norepinephrine group was 24.30 ± 2.44. Neonatal outcomes assessed in the form of Apgar scores at one and five minutes were similar in both groups and statistically insignificant (p > 0.05). No neonate had an Apgar score of less than 8 in either of the study groups (Table 3).

**Figure 2:**
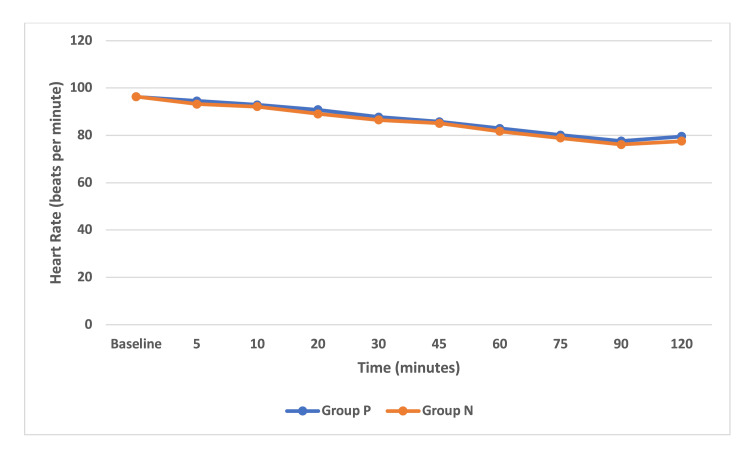
Intraoperative serial changes in heart rate. Data are represented as mean and are shown for 120 minutes.

A comparison of hemodynamic data was done between group P and group N with mean values at various time points by Student’s unpaired t-test. The mean HRe difference between the groups was not statistically significant (p > 0.05) at the various time intervals. The mean HR in group P ranged between 77 and 96 beats/minute and in group N ranged between 76 and 96 beats/minute. The difference in mean HR between the two groups was found to be similar at all time intervals (Figure 2).

**Figure 3:**
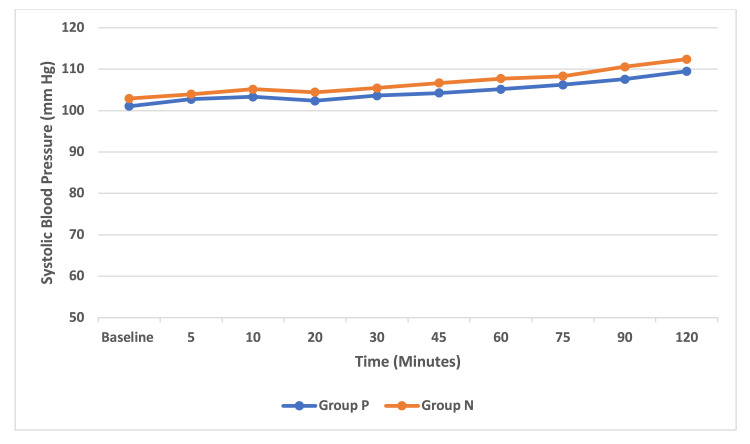
Intraoperative serial changes in systolic blood pressure. Data are represented as mean and are shown for 120 minutes.

**Figure 4:**
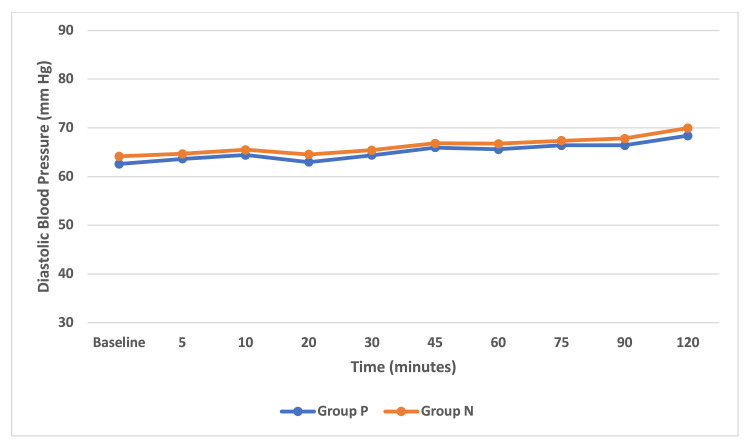
Intraoperative serial changes in diastolic blood pressure. Data are represented as mean and are shown for 120 minutes.

The mean SBP difference between groups was statistically significant (p < 0.05) from 20 minutes onwards up to 120 minutes which indicated that patients receiving norepinephrine infusion had higher mean SBP than the phenylephrine group. The mean SBP in group P was between 107 and 101 mmHg and in group N was between 110 and 102 mmHg (Figure 3). However, the comparison of mean DBP between the groups was not statistically significant (p > 0.05). Both the phenylephrine and norepinephrine groups were comparable. The mean DBP in group P was 66-62 mmHg and in group N was 67-64 mmHg (Figure 4). Within the groups, analysis for HR, SBP, and DBP with repeated-measures ANOVA with a Greenhouse-Geisser correction differed statistically significantly between time points (p < 0.05) for both group P and group N.

## Discussion

In our study, we assessed the total number of rescue bolus doses of phenylephrine needed to maintain SBP. The rescue bolus dose of phenylephrine 20 μg was given once SBP was less than 20% of the baseline value. In this study, in the phenylephrine group (group P), 34.6% of patients required one rescue bolus of phenylephrine to maintain SBP, and 12.5% of patients required two rescue boluses of phenylephrine. In group norepinephrine (group n), 22.1% of patients required one rescue bolus of phenylephrine after starting norepinephrine infusion and none of them required two rescue boluses of phenylephrine, and this difference was statistically significant (p < 0.005). Hence, the norepinephrine infusion group patients maintained a stable intraoperative blood pressure when compared to the phenylephrine infusion and were superior. This finding is similar to the studies conducted by Berawala et al. [19] and Goel et al. [20].

We measured the SBP at various time intervals between the norepinephrine and phenylephrine groups. Until 20 minutes from the start of the infusion, the mean SBP was comparable between the norepinephrine group and the phenylephrine group. After 20 minutes, patients in group N maintained a mean SBP at a higher level with a lesser requirement of rescue phenylephrine boluses compared to patients in the phenylephrine group (p-value lesser than 0.05). This trend was noted till the end of the procedure probably because of a lower dose of fixed-rate phenylephrine infusion.

Our study outcomes were similar to that conducted by Vallejo et al. [18] who compared fixed-rate infusions of phenylephrine and norepinephrine. Although they found no difference between groups in the proportion of patients who required rescue vasopressor boluses, patients in the phenylephrine group required more than one rescue dose of ephedrine (p < 0.01), and the incidence of emesis was greater in the phenylephrine group (p < 0.001). Hence, they concluded that norepinephrine fixed-rate infusion was more effective for preventing maternal hypotension.

Bradycardia in the phenylephrine group is due to its pure adrenergic agonist properties which have a dose-related propensity to decrease HR and CO. We did not observe any increased frequency of bradycardia, as seen in many other similar studies [20,21]. Moreover, we did not observe any other detrimental complications such as maternal shivering, nausea, and vomiting with our study drugs.

Our results also showed that norepinephrine had similar efficacy to phenylephrine without any detrimental neonatal outcomes when measured in terms of neonatal umbilical venous blood gas analysis and Apgar scores and was consistent with the study done by Singh et al. [17]. Maternal hypotension is a main factor affecting neonatal outcomes. As blood pressure was maintained within reasonable limits in both groups, the neonatal outcome was good in both groups.

Our results also agreed with the studies conducted by Puthenveettil et al. [6] and Sharkey et al. [15] who compared the intermittent bolus regimens of phenylephrine and norepinephrine to prevent and treat post-spinal maternal hypotension during cesarean delivery. They concluded that the hemodynamic profile offered by norepinephrine was superior to that of phenylephrine due to fewer fluctuations in HR and possibly CO. The number of norepinephrine boluses required to maintain blood pressure was much lesser than phenylephrine boluses. The neonatal arterial blood gases and Apgar scores were comparable without any adverse outcomes.

We also did not observe any hypertensive episodes in any of our groups requiring discontinuation of the vasopressor infusion and patient exclusion from the study.

In addition, there were no episodes of bradycardia associated with hypotension warranting the cessation of vasopressor infusion or the need for emergent administration of ephedrine 6 mg or atropine 0.6 mg and patient exclusion from our study.

Traditionally, ephedrine was regarded as the first choice drug to maintain maternal blood pressure. However, its tendency to cause fetal acidosis and repeated administration caused tachyphylaxis. Therefore, it became the preferred second-line drug for the management of post-spinal hypotension according to a recent consensus statement [22].

Phenylephrine became the gold standard drug of choice in obstetric patients among the various vasopressors used to treat post-spinal maternal hypotension [10]. However, its complications included reflex bradycardia and decreased CO [5]. Hence, norepinephrine which has a weak β-adrenergic receptor agonist activity in addition to potent α-adrenergic receptor activity was recently introduced for prophylaxis against post-spinal hypotension during cesarean delivery as a promising alternative to ephedrine and phenylephrine. Ngan Kee et al. first used a norepinephrine infusion of 5 μg/mL and found it to be efficient in maintaining blood pressure without any adverse neonatal outcomes [12]. Prophylactic infusions of norepinephrine were also used to maintain maternal blood pressure without any adverse neonatal outcomes [13].

Vasopressors are usually given as intermittent boluses or as infusions, but infusions allow titratable blood pressure control with fewer manual interventions by the anesthetist in hemodynamic management [9].

Hasanin et al. [23] compared three doses of norepinephrine infusion during cesarean delivery to determine the optimum dose. The two higher doses of 0.050 μg/kg/minute and 0.075 μg/kg/minute were equally effective in decreasing the frequency of post-spinal hypotension compared with a lower dose of 0.025 μg/kg/minute. No further advantage was found for delivering the higher-dose infusion of 0.075 μg/kg/minute compared with the 0.050 μg/kg/minute infusion, suggesting that 0.050 μg/kg/minute is the best dose for norepinephrine during cesarean delivery which we chose for our study. Another benefit of norepinephrine is that it is more cost-effective than phenylephrine.

As reported in previous studies, we found that norepinephrine can be safely administered through peripheral veins when given through a large-bore catheter and provided that it was diluted [13,14]. We observed no extravasation of the vasopressor infusion or any paleness of the skin at the site of infusion in any group.

Most studies have stopped the infusion of both vasopressors after delivery. Abrupt termination of vasopressor infusion can lead to episodes of hypotension as the autonomic spinal blockade is persistent even after delivery which can continue to cause hypotension. Moreover, oxytocin infusion after delivery can decrease maternal blood pressure due to peripheral vasodilatation. Additionally, blood loss due to various causes such as longer duration of the procedure, adhesions, and atonic postpartum hemorrhage can also contribute to maternal hypotension. Hence, we continued the infusions keeping in mind that the concentration of the vasopressor solutions which we used was very low.

This study had a few limitations. We did not use any advanced cardiac monitors for CO measurement as inserting invasive arterial lines is not an acceptable practice for uncomplicated cesarean deliveries. The non-invasive blood pressure recordings may not be precisely timed when rescue boluses were administered and may be prone to artifacts. The applicability of this study in emergency cesarean sections needs to be studied. Norepinephrine rescue bolus protocols were recently reported in obstetric anesthesia but we did not follow them. The decision for using a fixed rate versus a titrated rate for vasopressor infusion during cesarean delivery is controversial. Further clinical research is recommended before adapting to obstetric anesthesia practice. The possible hemodynamic fluctuations induced by oxytocin and interventions for the same should have been analyzed. Further studies need to be performed in parturients with cardiac morbidities, severe pre-eclampsia, or conditions of reduced uteroplacental flow to determine the applicability of these results to this specific obstetric population.

## Conclusions

Prophylactic fixed-rate norepinephrine infusion when compared to prophylactic fixed-rate phenylephrine infusion was associated with a lesser requirement of rescue phenylephrine boluses and effectively reduced post-spinal maternal hypotension in cesarean deliveries. The hemodynamic variations and neonatal outcomes were similar in both groups. Hence, norepinephrine infusion is as effective as phenylephrine for maintaining blood pressure after spinal anesthesia in cesarean delivery with a stable HR and can be considered as an alternative in pregnant women in whom phenylephrine has to be used with caution such as those with lower HRs and uteroplacental insufficiency.

## References

[1] Heesen M, Stewart A, Fernando R (2015). Vasopressors for the treatment of maternal hypotension following spinal anaesthesia for elective caesarean section: past, present and future. Anaesthesia.

[2] Hasanin A, Aiyad A, Elsakka A (2017). Leg elevation decreases the incidence of post-spinal hypotension in cesarean section: a randomized controlled trial. BMC Anesthesiol.

[3] Hasanin A, Soryal R, Kaddah T (2018). Hemodynamic effects of lateral tilt before and after spinal anesthesia during cesarean delivery: an observational study. BMC Anesthesiol.

[4] Veeser M, Hofmann T, Roth R, Klöhr S, Rossaint R, Heesen M (2012). Vasopressors for the management of hypotension after spinal anesthesia for elective caesarean section. Systematic review and cumulative meta-analysis. Acta Anaesthesiol Scand.

[5] Hasanin A, Mokhtar AM, Badawy AA, Fouad R (2017). Post-spinal anesthesia hypotension during cesarean delivery, a review article. Egypt J Anaesth.

[6] Puthenveettil N, Sivachalam SN, Rajan S, Paul J, Kumar L (2019). Comparison of norepinephrine and phenylephrine boluses for the treatment of hypotension during spinal anaesthesia for caesarean section - a randomised controlled trial. Indian J Anaesth.

[7] Carvalho B, Dyer RA (2015). Norepinephrine for spinal hypotension during cesarean delivery: another paradigm shift?. Anesthesiology.

[8] Mets B (2016). Should norepinephrine, rather than phenylephrine, be considered the primary vasopressor in anesthetic practice?. Anesth Analg.

[9] Choudhary M, Bajaj JK (2018). Study comparing phenylephrine bolus and infusion for maternal hypotension and neonatal outcome during cesarean section under spinal anesthesia. Anesth Essays Res.

[10] Ngan Kee WD (2017). The use of vasopressors during spinal anaesthesia for caesarean section. Curr Opin Anaesthesiol.

[11] Ngan Kee WD (2017). A random-allocation graded dose-response study of norepinephrine and phenylephrine for treating hypotension during spinal anesthesia for cesarean delivery. Anesthesiology.

[12] Ngan Kee WD, Lee SW, Ng FF, Tan PE, Khaw KS (2015). Randomized double-blinded comparison of norepinephrine and phenylephrine for maintenance of blood pressure during spinal anesthesia for cesarean delivery. Anesthesiology.

[13] Ngan Kee WD, Lee SW, Ng FF, Khaw KS (2018). Prophylactic norepinephrine infusion for preventing hypotension during spinal anesthesia for cesarean delivery. Anesth Analg.

[14] Onwochei DN, Ngan Kee WD, Fung L, Downey K, Ye XY, Carvalho JC (2017). Norepinephrine intermittent intravenous boluses to prevent hypotension during spinal anesthesia for cesarean delivery: a sequential allocation dose-finding study. Anesth Analg.

[15] Sharkey AM, Siddiqui N, Downey K, Ye XY, Guevara J, Carvalho JC (2019). Comparison of intermittent intravenous boluses of phenylephrine and norepinephrine to prevent and treat spinal-induced hypotension in cesarean deliveries: randomized controlled trial. Anesth Analg.

[16] Sjöberg T, Norgren L, Andersson KE, Steen S (1989). Comparative effects of the alpha-adrenoceptor agonists noradrenaline, phenylephrine and clonidine in the human saphenous vein in vivo and in vitro. Acta Physiol Scand.

[17] Singh J, Singh J, Mitra S, Anand LK, Goel B, Kaur M (2022). Comparison of prophylactic phenylephrine and norepinephrine infusion on umbilical arterial pH and maternal blood pressure during spinal anaesthesia for caesarean delivery. Indian J Anaesth.

[18] Vallejo MC, Attaallah AF, Elzamzamy OM (2017). An open-label randomized controlled clinical trial for comparison of continuous phenylephrine versus norepinephrine infusion in prevention of spinal hypotension during cesarean delivery. Int J Obstet Anesth.

[19] Berawala PK, Mehta SH, Chaudhari MS, Shinde MK (2021). A randomized, double-blinded comparative study of phenylephrine infusion and norepinephrine infusion for the prevention and treatment of spinal anesthesia-induced hypotension in elective and emergency cesarean deliveries. Indian Anaesth Forum.

[20] Goel K, Luthra N, Goyal N, Grewal A, Taneja A (2021). Comparison of norepinephrine and phenylephrine infusions for maintenance of haemodynamics following subarachnoid block in lower segment caeserean section. Indian J Anaesth.

[21] Chen Z, Zhou J, Wan L, Huang H (2022). Norepinephrine versus phenylephrine infusion for preventing postspinal hypotension during cesarean section for twin pregnancy: a double-blinded randomized controlled clinical trial. BMC Anesthesiol.

[22] Kinsella SM, Carvalho B, Dyer RA (2018). International consensus statement on the management of hypotension with vasopressors during caesarean section under spinal anaesthesia. Anaesthesia.

[23] Hasanin AM, Amin SM, Agiza NA (2019). Norepinephrine infusion for preventing postspinal anesthesia hypotension during cesarean delivery: a randomized dose-finding trial. Anesthesiology.

